# Massively parallel direct writing of nanoapertures using multi-optical probes and super-resolution near-fields

**DOI:** 10.1038/s41378-022-00416-9

**Published:** 2022-09-15

**Authors:** Changsu Park, Soobin Hwang, Donghyun Kim, Nahyun Won, Runjia Han, Seonghyeon Jeon, Wooyoung Shim, Jiseok Lim, Chulmin Joo, Shinill Kang

**Affiliations:** 1 School of Mechanical Engineering, Yonsei University, 50 Yonsei-ro, Seodaemun-gu, Seoul, 03722 Korea; 2 National Center for Optically-Assisted High Precision Mechanical Systems, Yonsei University, Seoul, 03722 Korea; 3 Department of Materials Science and Engineering, Yonsei University, 50 Yonsei-ro, Seodaemun-gu, Seoul, 03722 Korea; 4 School of Mechanical Engineering, Yeungnam University, 280 Daehak-ro, Gyeongsan, Gyungbuk 38541 South Korea

**Keywords:** Optical materials and structures, Micro-optics, Other nanotechnology

## Abstract

Laser direct-writing enables micro and nanoscale patterning, and is thus widely used for cutting-edge research and industrial applications. Various nanolithography methods, such as near-field, plasmonic, and scanning-probe lithography, are gaining increasing attention because they enable fabrication of high-resolution nanopatterns that are much smaller than the wavelength of light. However, conventional methods are limited by low throughput and scalability, and tend to use electron beams or focused-ion beams to create nanostructures. In this study, we developed a procedure for massively parallel direct writing of nanoapertures using a multi-optical probe system and super-resolution near-fields. A glass micro-Fresnel zone plate array, which is an ultra-precision far-field optical system, was designed and fabricated as the multi-optical probe system. As a chalcogenide phase-change material (PCM), multiple layers of Sb_65_Se_35_ were used to generate the super-resolution near-field effect. A nanoaperture was fabricated through direct laser writing on a large-area (200 × 200 mm^2^) multi-layered PCM. A photoresist nanopattern was fabricated on an 8-inch wafer via near-field nanolithography using the developed nanoaperture and an i-line commercial exposure system. Unlike other methods, this technique allows high-throughput large-area nanolithography and overcomes the gap-control issue between the probe array and the patterning surface.

## Introduction

The most promising method to achieve fine patterns for semiconductors, displays, biosensors, and other micro/nanodevices is to use laser direct writing and nanolithography^1–4^. Meanwhile, electronics miniaturization and high-density integration have been studied to develop new technologies^5^. Lithography is an important process for high-density integration; however, it is expensive and time-consuming, and semiconductor manufacturing requires a large number of lithography-related processes.

The traditional photolithography process is based on the resolution equation described in the Rayleigh criterion, in which line width is reduced using a large lens aperture and short-wavelength light sources^1^. It is difficult to apply this process to high-variety low-volume production owing to the high costs related to design and production, which may vary depending on the model, as well as subsequent maintenance and management costs that are incurred due to the production of masks^6^. Additionally, laser light is projected onto a substrate via projection optics to produce high-precision patterns; this process requires highly expensive light sources with short wavelengths and high-magnification optics for high-resolution patterning. Maskless lithography based on direct-writing technology can generate arbitrary shapes and is therefore appropriate for low-volume production of nanopatterns with arbitrary features. However, the wavelength of the light source and the numerical aperture of the lens limit the patterning resolution owing to light diffraction, which in turn limits application of this technology to high-precision sub-wavelength patterning. To commercialize maskless lithography, methods to achieve high-resolution high-throughput patterning are required.

To overcome the aforementioned disadvantages of conventional photolithography, several direct-writing and lithography techniques have been studied, such as focused-ion-beam patterning, e-beam lithography, scanning-probe lithography and plasmonic lithography^7–13^. Light localization at the sub-wavelength scale can be obtained by passing light through a pinhole with a diameter smaller than the wavelength of the light; therefore, nanoscale patterning with a near-field light source is used in near-field lithography. However, pinholes with small diameters produce a less intense light source, which can reduce patterning speed. To address the limitations of near-field lithography, two methods have been proposed: parallel patterning to increase the patterning area, and the use of surface plasmons to increase light transmittance through the pinhole. The Graham J. Leggett group proposed scanning near-field-optical-microscopy patterning, in which scanning near-field optical probes are assembled in parallel to generate patterns. They achieved a resolution of 70 nm using 12 probes; however, the patterning speed was slow (0.2 µm/s)^14^. The Chad. A. Mirkin group presented nanoscale digital beam pen lithography based on a digital mirror device. They designed 15,000 pyramid probes per 1 cm^2^ using a flexible material (polydimethylsiloxane)^8,9^. The Xiang Zhang group designed aerodynamic flying plasmonic lenses that could maintain a set distance (such as ~20 nm) above the surface of a hard disk. These lenses successfully produced a pattern of ~80 nm at high speeds of 4–12 m/s per second. Contact plasmonic nanolithography technology, which improves current recording speeds by hundreds of times, has been reported^11^. This technology uses a near field from a contact probe coated with a self-assembled monolayer and a protective diamond-like carbon layer, and supports parallel patterning; moreover, a pattern size of less than 50 nm and a maximum patterning speed of 10 mm/s can be achieved.

Super-resolution near-field structures (super-RENSs) using a phase-change material (PCM) function as optical dynamic apertures that can be used to fabricate nanopatterns via reducing the effective size of the laser beam to smaller than the diffraction limit by exploiting thermal and optical characteristics and the reaction of the PCM with the incident beam^15,16^. Research on super-resolution near-field structures has primarily been conducted by Junji Tominaga, A. C. Assafrao, and Jingsong Wei to improve storage capacity in the optical memory field^17–22^. In this technique, a thin layer of a PCM is covered with a dielectric layer on the top and bottom sides in an optical disk stack layer. Super-RENSs include the aperture-type (transparent type) using chalcogenide PCMs such as antimony (Sb), germanium antimony telluride (GeSbTe), antimony telluride (SbTe), and silver indium antimony telluride (AgInSbTe) for enhanced local permeability^23–26^; and light-scattering center types through local plasmon, which are formed through reversible silver oxide (AgOx) and platinum oxide (PtOx) decomposition^27,28^. By radiating a laser beam, PtOx is decomposed at high temperatures into platinum nanoparticles and oxygen. Junji Tominaga studied the Sb-based nanolithography process^28^. This process focused on the fabrication of a nanopattern on the bottom of an optical disk, which is not a conventional patterning substrate such as silicon wafer and other substrates.

However, some critical drawbacks of near-field-lithography technologies remain unaddressed, such as the need for ultra-precision gap control, highly expensive production, and long production times caused by low scan rates and poor transmittance efficiency. Therefore, in this study, we performed massively parallel direct-writing using a glass Fresnel zone plate (FZP) array as an ultra-precision far-field micro-optical system. A multi-optical probe system was fabricated through CO_2_ laser-assisted glass imprinting, as proposed in our previous study^29^. Furthermore, we developed a multi-layered PCM using antimony selenide (Sb_65_Se_35_) and zinc sulfide-silicon dioxide (ZnS-SiO_2_) with a super-resolution near-field effect. We also developed nanoapertures through massively parallel direct writing for use in an i-line commercial exposure system. To verify the effectiveness of the writing process, we designed a photoresist nanopattern using near-field lithography.

## Results and discussion

### Concept of massively parallel direct writing of nanoapertures

In this study, nanoapertures were created using a multi-layered PCM and a glass FZP array equipped with a multi-optical probe system to achieve near-field super-resolution patterning. Generally, in direct laser writing, patterning precision is related to the spot size of the beam focal point, which is in turn related to the numerical aperture of the objective lens and the laser wavelength. To improve patterning precision, researchers have attempted to use light sources with shorter wavelengths and to increase the numerical aperture of the lens. However, the diffraction of light fundamentally limits patterning precision in far-field optics; these systems irradiate direct light through an objective lens. Therefore, to overcome the diffraction limit, solid immersion lens technologies, based on near-field optical systems, and plasmonics-based technologies have been investigated. However, these technologies do not eliminate the problems of high processing costs and long processing times resulting from low scan rates and the precise gap control required to ensure a distance of less than 10 nm between the optical system and the photosensitive material. Therefore, we suggest the use of an ultra-precision nanoaperture designed using our proposed super-resolution near-field optical structure. Through this technique, a far-field-based optical system can be utilized for near-field optics applications.

**Fig. 1:**
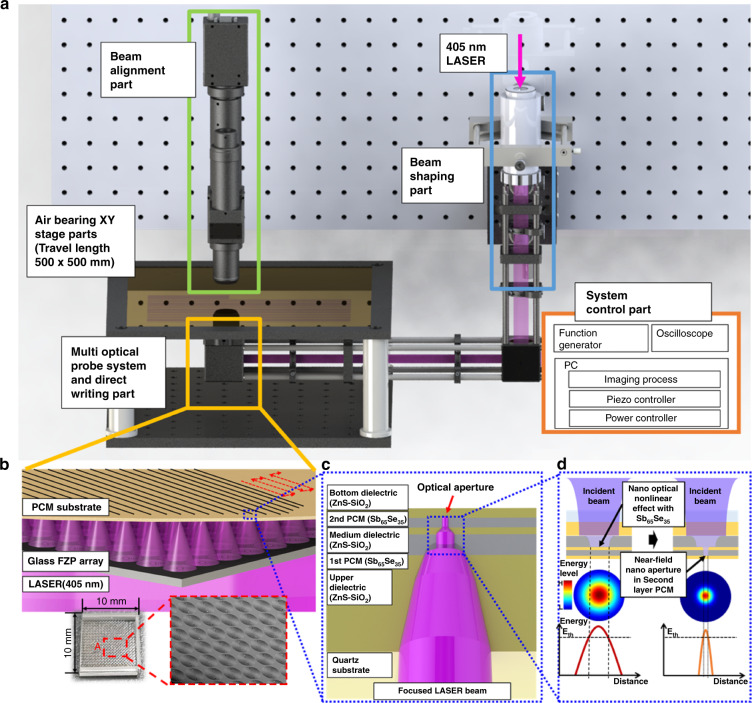
Concept of massively parallel direct writing of nanoapertures using a multi-optical probe system and super-resolution near-fields. **a** Schematic of a massively parallel direct-writing system. **b** Direct-writing parts and multi-optical probe system using glass FZP array (Photo and SEM image 20 × 20 glass FZP array on K-PG375 glass of 10 × 10 mm^2^). **c** Schematic of multi-layered PCM for super-resolution near-field effect. In the multi-layered PCM, the PCM layers are Sb_65_Se_35_ and the dielectric layers are composed of ZnS-SiO_2_. The upper dielectric layer is 180 nm thick, the first PCM layer is 20 nm thick, the medium dielectric layer is 5 nm thick, the second PCM layer is 10 nm thick, and the bottom dielectric layer is 5 nm thick. **d** Schematic of the direct-writing process using the nano-optical nonlinear effect with Sb_65_Se_35_. When the laser is focused on the first PCM layer, the absorbed power is converted into heat; this creates a nano-optical aperture, which generates a nanofocused beam that generates a sub-diffraction nanoaperture on the second PCM layer

Figure 1 illustrates a massively parallel direct-writing process involving a multi-optical probe system and a super-resolution near-field effect. A laser light source is directed to the first PCM layer (Sb_65_Se_35_) to trigger a super-resolution near-field effect, and nanoaperture patterning is induced in the second PCM layer. After passing through the first dielectric layer, the beam has a smaller spot size due to the higher refractive index compared with quartz. The penetrating beam reaches the first PCM layer and is absorbed in the PCM based on its high absorption coefficient. The absorbed energy is converted into heat, and this increases the temperature of the PCM. Here, the crystalline phase of the Sb_65_Se_35_ transforms into a localized amorphous phase at 853 K, the threshold temperature of Sb_65_Se_35_. In addition, beam focusing via the thermally induced optical nonlinear effect occurs on the boundary surface between the crystalline and amorphous phases^30^. This localized beam is transmitted locally owing to its high optical penetration rate in the amorphous phase of Sb_65_Se_35_. The nanofocused beam passes through the second dielectric layer and then the second Sb_65_Se_35_ PCM layer, to generate an optical aperture smaller than the light focus size (due to additional localized heating), as with the first PCM layer. At this point, the dielectric layers play the role of preventing the heating of the PCM as a result of the high power during laser irradiation. In addition, the second dielectric layer maintains a set gap between the first and second PCM layers, which aids in generating a stable and uniform size of optical aperture. Finally, the third dielectric layer creates a gap between the PCM and the photoresist during the lithography process, which aids in forming resist patterns with uniform line widths.

### Design methodology of a multi-probe optical system: Glass FZP array

An FZP is an optical device with several concentric rings; it focuses light by diffracting transmitted light, generating interference in the focal plane. There are two types of FZP: the amplitude type, which generates diffraction and interference through successive arrays of transparent and opaque rings, and the phase type, in which alternating zones generate different phase shifts, causing interference. The phase type is composed of a single material. To achieve maximum design efficiency, the phase shift between two levels must be *π*^31^.

The FZP zone radius can be calculated from the Pythagorean theorem using the optical path and first-order focal length. The radius of an nth zone is expressed as:

1 $$r_n = \sqrt {n\lambda f + \left( {n\frac{\lambda }{2}} \right)^2}$$

where *ƒ* is the focal length of the Fresnel zone plate, *λ* is the wavelength of the light source to be used for focusing, and $r_n$ is the radius of the *n*th ring of the Fresnel zone plate to be manufactured. Therefore, the wavelength of the radiation, focal length, and minimum zone width are important parameters in FZP design. In this study, the laser wavelength used in the direct-writing system was 405 nm, the required focal length was 1000 μm, the minimum zone width was 1 μm, and the radius of the outermost ring was 200 μm. To confirm the characteristics of the designed FZP, we simulated free-space beam propagation using MATLAB based on the Fresnel–Kirchhoff diffraction theory^32^.

**Fig. 2:**
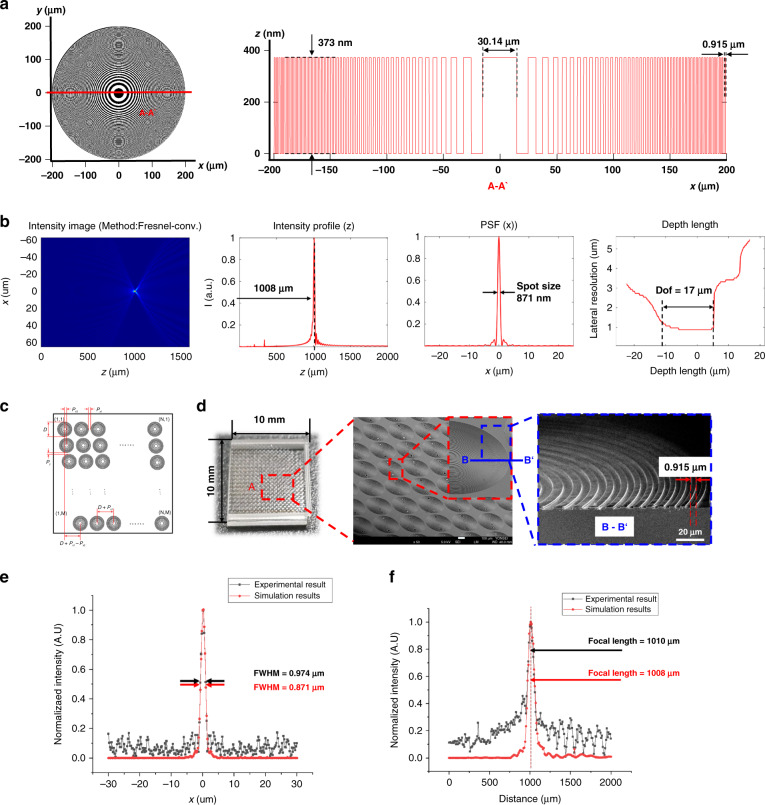
Design and fabrication results for a glass FZP array. **a** Top view of an FZP. **b** The FWHM value of the intensity distribution at the focal plane of the designed FZP lens. **c** Glass FZP array strategy. *D* = 400 μm, *M* x *N* is a 20 × 20 array, $P_{x1}$ = 20 μm, $P_{x2}$ = 21 μm, $P_y$ = 25 μm. **d** SEM image of the FZP array fabricated using K-PG375 glass imprinted using a glass thermal-imprinting system that included an infrared laser. **e** The simulation and experimental FWHM values of the average beam intensities of the glass FZP. **f** The simulated and experimental focal lengths of the glass FZP were compared by measuring normalized spot intensities

A glass FZP array was fabricated using a K-PG375 substrate with a refractive index of 1.5425. The FZP array had 49 rings, and the radius of the outermost ring was 200 μm. By simulating free-space beam propagation using MATLAB, we could predict whether an FZP array fabricated on a glass substrate would deliver optical performance suitable for the intended use. The wavelength of the light source was 405 nm, and the height of the pattern was set to 373 nm. The lateral resolution and depth of focus (DoF) were estimated by measuring the full width at half maximum (FWHM) of the lateral focus profile after Gaussian fitting. Figure 2a, b shows design and simulated results for a glass FZP array. A diameter of FZP is 400 μm, and a cross-sectional analysis of the FZP ring distribution at the A-A’ mark. It can be seen that the height of the FZP rings is 373 nm, the minimum line width of the ring is 0.915 μm, and 30.14 μm is the maximum line width. Based on MATLAB-based beam-propagation simulation (b) the FWHM value of the intensity distribution at the focal plane of the designed FZP lens is 915 nm; the FZP has a focal length of 1008 μm, and the DoF is 17 μm. The DoF value is defined as the axial length over which the size of the lateral beam spot is smaller than the square root of the minimum spot size.

To produce nanoapertures on a large-area (200 mm^2^) substrate, the glass FZP array was efficiently arranged. Parameters were defined for the arrangement of the FZP to control the width and pitch of the nanoapertures: the diameter of one FZP (*D*), the number of FZP columns (*N*), the number of FZP rows (*M*), the *x*-axis length of the glass FZP substrate (*X*), the *y*-axis length of the glass FZP substrate (*Y*), the *x*-axis spacing between (*n*,*m*) FZP and (*n* + 1, *m*) FZP ($P_{x1}$), and the *x*-axis spacing between (*n*, *m*) FZP and (*n*, *m* + 1) FZP ($P_{x2}$),

2 $$N \times \left( {{{D}} + P_{x1}} \right) - P_{x2} + D = X_{{\mathrm{FZP}}}\, < \,{{{\mathrm{X}}}}$$

3 $$\left( {M - 1} \right) \times \left( {{{D}} + P_{x1}} \right) + D = Y_{{\mathrm{FZP}}}\, < \,{{{\mathrm{Y}}}}$$

4 $${\mathrm{Number}}\;{\mathrm{of}}\;{\mathrm{scans}} = P_{x2}/{\mathrm{pattern}}\;{\mathrm{pitch}}$$

*M* x *N* was a 20 × 20 array, $P_{x1}$ was 20 µm, $P_{x2}$ was 21 µm, $P_y$ was 25 µm, $X_{{\mathrm{FZP}}}$ was 8.779 mm, and $Y_{{\mathrm{FZP}}}$ was 8.475 mm. The glass FZP array was designed to enable the pattern pitch to change according to the *x*-axis movement, and the condition of 10 ×10 mm is a specification requirement for glass-imprinting systems that can be manufactured according to the FZP design criteria. Figure 2d shows scanning electron microscopy images of the glass FZP array fabricated through infrared laser-based thermal imprinting on glass. The laser-assisted glass thermal-imprinting system and fabrication process are described in the Methods section. The optical performance of the glass FZP array was evaluated (Fig. 2e–f). The FWHM lateral spot size was measured to be 0.974 µm, which is approximately 11.3% greater than the theoretical value of 0.871 µm (standard deviation: 0.0463 µm). This discrepancy can be attributed to measurement error associated with the different sampling rates or DoF. We also analyzed the focal length (Fig. 2f). Beam intensities were measured when the distance between the FZP and focal plane of the objective lens mounted at the charge-coupled device was 0–2 mm. The measured values were compared with the simulation data. The experimental and theoretical focal lengths were 1008 µm and 1010 µm, respectively.

### Design methodology and optimization of multi-layered phase-change materials

Design optimization of a multi-layered PCM is essential to achieve the super-resolution near-field effect. Such an optimization process was applied in an earlier study of direct-writing nanolithography using a PCM^33^. That study presented an effective method for designing and optimizing dielectric layers and a PCM layer to obtain ultrafine patterns. In the current study, we determined the size of the optical aperture according to the thicknesses of the five layers that constituted the PCM. We also determined the required thickness of the layers to minimize the size and line width of the nanoapertures. To determine the laser power absorbed by the irradiated multi-layered PCM, optical analysis was performed using the rigorous coupled-wave approach. A two-dimensional (2D) linear transient conduction analysis based on the FEM provided information on the temperature of individual PCM layers according to the degree of laser beam absorption^34–37^. The optimal layer thickness was set according to the minimum aperture size. We analyzed the intensity of light transmitted to the PCM using the finite-difference time-domain method^38^.

First, rigorous coupled-wave analysis (RCWA) was applied to analyze the laser light transmitted through the multi-layered PCM. Based on Maxwell’s equation, the FMM is applied to the laser beam in the form of a Gaussian function as an input electric field. It is expressed as:

5 $$\left| E \right| \cdot {\mathrm{exp}}\left( { - \frac{{x^2 + y^2}}{{2 \cdot \sigma ^2}}} \right)$$

where |E| is the magnitude of the electric field and *σ* is the deviation in the Gaussian distribution, which indicates the FWHM of the laser beam. The intensity can be calculated as:

6 $${{{\mathrm{I}}}} = \frac{{cn\varepsilon _0}}{2}\left| E \right|^2$$

where *I* is the light intensity, *c* is the velocity of light, *n* is the refractive index of the material, and $\varepsilon _0$ is the permittivity of free space. The light intensity is given by

7 $${{{\mathrm{I}}}} = \frac{P}{{\pi \cdot {{\Phi }}^2}}$$

where *P* is the laser power and *Φ* is the minimum beam spot size according to the resolution equation ($\lambda /\pi \cdot {\mathrm{NA}}$). These equations can be used to calculate the electric and magnetic fields in the dielectric and PCM layers. Ultimately, the power flow can be obtained through the vector product of the two fields. The absorbed power per unit volume in the current step can be solved using the power flow obtained in the previous step, and the equation used is as follows:

8 $${{Q}}\left( {{{x}},{{y}}} \right) = \frac{{S_z^{n - 1} - S_z^n}}{{{\mathrm{dz}}}}$$

where *Q(x, y)* is the power per unit volume and $s_z$ is the power flow per unit area in the *z*-direction. In the actual simulations, the step size (dz) was set to 1 nm.

Using the above-mentioned equations, the power flow of the Gaussian beam penetrating the multi-layered PCM can be analyzed. In addition, by confirming the level of power absorption indicated by the results, thermal energy and temperature information for the PCM can be obtained. The 2D linear transient thermal conduction equation was used to analyze the heat transfer of the absorbed optical energy^34–36^:

9 $$\rho C_p\frac{{\partial T}}{{\partial t}} - \kappa \left( {\frac{{\partial ^2T}}{{\partial x^2}} + \frac{{\partial ^2T}}{{\partial y^2}}} \right) = \bar Q(x,y) \cdot G(t)$$

where *ρ* is the material density, $C_p$ is the specific heat, *κ* is the thermal conductivity, and $\bar Q(x,y)$ is the average power per unit time and unit volume at a specific position on the PCM layer. The spatial step was 5 nm in the *x* and *y* directions and the time step was 10 ns. Antimony chalcogenide series materials are characterized by a rapid change in their absorption coefficient with phase change. Thus, the reset time, which is the transition time required for a crystalline structure to transform into an amorphous structure, was set according to the time-domain analysis in the model configuration, with the time ranging from several to dozens of nanoseconds. In this study, we set the time domain to 35 ns, which is the reset time of Sb_65_Se_35_^31^. According to the resolution equation ($\lambda /\pi \cdot {\mathrm{NA}}$), the beam spot where the laser irradiates the PCM layer is very local, and the distance from the end of the layer is very large. Thus, an adiabatic boundary condition was set. Moreover, the area surrounding the spot was set as a finite reservoir, and diffusion in the *x* and *y* directions was assumed. The equation for $G(t)$ below accounts for the pulsed mode of the Gaussian beam, as follows:

9 $$G\left( t \right) = {\mathrm{exp}}\left( { - \frac{{t^2}}{{\varsigma ^2}}} \right),\left( {0\, < \,t\, < \,\frac{1}{{{\mathrm{Hz}}}}} \right)$$

where $\varsigma$ is the FWHM of the laser pulse width. The first and second PCM layers are a few nanometers thick. Thermal analysis in the *z*-direction of the Gaussian beam considered thermal conduction only in the *x* and *y* directions of a 2D plane.

The 2D linear transient conduction equation was solved using 2D FEM analysis to generate the thermal distribution in the PCM layers. Our results confirm that only localized heating occurred at 853 K (the melting temperature of Sb_65_Se_35_), with a phase transition from the crystalline to the amorphous phase^33^. The aperture opening in the first PCM layer was determined accordingly and then used to obtain the final aperture size in the second PCM layer.

**Fig. 3:**
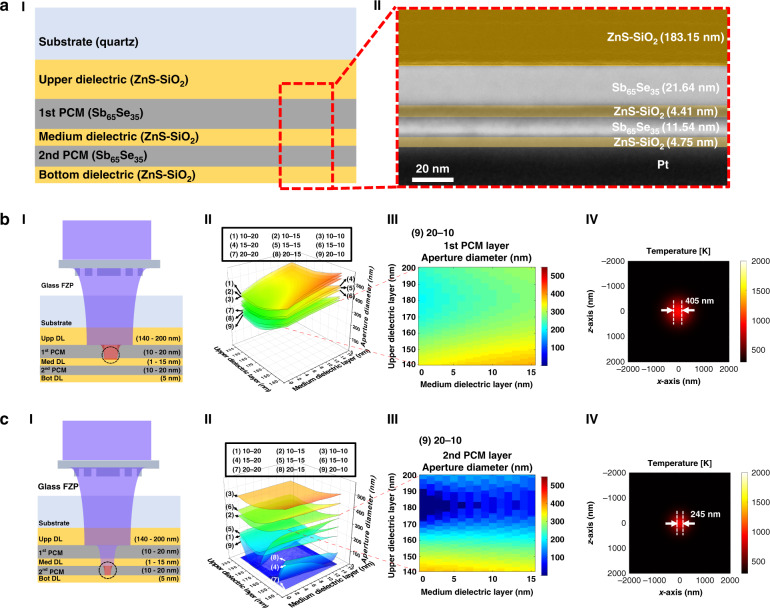
Optimization of multi-layered PCM for the super-resolution near-field effect. **a** I. Schematic of multi-layered PCM for the super-resolution near-field effect. II. TEM image of the optimized and deposited multi-layered PCM, in which the PCM and dielectric layers were composed of Sb_65_Se_35_ and ZnS-SiO_2_. The upper dielectric layer was 183.15 nm thick, the first PCM layer was 21.64 nm thick, the medium dielectric layer was 4.41 nm thick, the second PCM layer was 11.54 nm thick, and the bottom dielectric layer was 4.75 thick. **b** I. Schematic of multi-layered PCM and aperture formation on the 1st PCM layer. II. Contour plots of aperture size on the first PCM layer according to the thickness of the upper dielectric layer (140–200 nm) and the medium dielectric layer (5–15 nm) for varying first and second phase-change layer thicknesses (1) 10–20 nm, (2) 10–15 nm, (3) 10–10 nm, (4) 15–20 nm, (5) 15–15 nm, (6) 15–10 nm, (7) 20–20 nm, (8) 20–15 nm, and (9) 20–10 nm. II. Contour plots of the aperture size on the first PCM layer with optimized PCM layer thicknesses of 20–10 nm (I–9) (other plots are available in Supplementary Information I-[1–8].) III. Figure S2(a)) IV. Temperature profile on the first PCM layer and aperture-prediction results. **c** I. Schematic of multi-layered PCM and aperture formation on the second PCM layer. II. Contour plots of the aperture size on the second PCM layer according to the thicknesses of the upper (140–200 nm) and medium dielectric layers (5–15 nm) and first and second PCM layers: (1) 10–20 nm, (2) 10–15 nm, (3) 10–10 nm, (4) 15–20 nm, (5) 15–15 nm, (6) 15–10 nm, (7) 20–20 nm, (8) 20–15 nm, and (9) 20–10 nm III. Contour plots of the aperture size on the second PCM layer with optimized thicknesses of 20–10 nm (II-9) (other plots are available in Supplementary Information II-[1–8].) IV. Temperature profile of the second PCM layer and aperture-prediction results

Figure 3a shows a schematic and a TEM image of the optimized multi-layered PCM for the super-resolution near-field effect. In the multi-layered PCM, the PCM and dielectric layers are composed of Sb_65_Se_35_ and ZnS-SiO_2_. The upper dielectric layer is 183.15 nm thick, the first PCM layer is 21.64 nm thick, the medium dielectric layer is 4.41 nm thick, the second PCM layer is 11.54 nm thick, and the bottom dielectric layer is 4.75 nm thick. Figure 3b shows the size of the optical aperture in the first PCM layer according to the thicknesses of the first and second dielectric layers and the first and second PCM layers, under a pulsed laser power of 100 mW, a repetition rate of 500 kHz, and a length of 20 ns. The thinner bottom dielectric layer allowed effective transmission of light to the photoresist through the opening. The transient conduction analysis was based on a fixed thickness of 5 nm, which could be controlled during the actual process. The analysis showed that a thinner dielectric layer led to a smaller optical aperture. Furthermore, an upper dielectric layer with a thickness of ~180 nm formed the smallest spot size; the thicker the first PCM layer, the smaller the aperture formed on the layer. A thicker PCM absorbs less laser power in the *z*-direction, which leads to the formation of a smaller aperture. The size of the optical aperture in the second PCM layer (Fig. 3c), also obtained through transient conduction analysis, was similar to that of the first PCM layer. The 180-nm-thick upper dielectric layer corresponded to the minimum aperture size. Thus, the thickness of the first PCM layer had a greater influence on the aperture size than did the thickness of the second PCM layer. Furthermore, the thickness of both PCM layers must be appropriate to minimize the aperture size. The optimized configuration was a 180-nm-thick upper layer, a 20-nm-thick first PCM layer, a 5-nm-thick medium dielectric layer, and a 10-nm-thick second PCM layer. Accordingly, we obtained minimum aperture sizes of 405 and 245 nm on the first and second PCM layers, respectively. Further information on the contour plots for first and second PCM layers of different thicknesses is described in the Supplementary Information (Fig. S2).

### Reasons for choosing Sb_65_Se_35_ over other materials as the PCM layer

PCM undergoes significant changes in physical properties, including optical, thermal, and electrical characteristics, with a transition between crystalline and amorphous phases^30^. Optical properties tend to exhibit the greatest changes. As shown in Table 1, both the refractive index (*n*) and extinction coefficient (*k*) varied greatly with the material phase. Compared with other materials, Sb_65_Se_35_ exhibits larger differences in optical properties between phases. Thus, Sb_65_Se_35_ is suitable as a multi-layered PCM, as it can form optical nanoapertures in lithography operations. In addition, Sb_65_Se_35_ has a low melting temperature, high thermal conductivity, and low-phase reset time. To verify these characteristics, the optimal PCM conditions to minimize the aperture size were identified through photothermal simulations. (Fig. S3)

**Table 1:** Materials property of phase-change materials (Ge_2_Sb_2_Te_5,_ Sb_2_Te_3,_ Sb_65_Se_35_)^30,31,39–42^

	Ge_2_Sb_2_Te_5_		Sb_2_Te_3_		Sb_65_Se_35_	
Melting Temperature (K)	893		893		853	
Mass density (kg/m^3^)	6350		6500		5653	
Specific heat per unit mass (J/kgK)	250		280		234	
Thermal conductivity (J/mks)	1.48		2.1		2.575	
Refractive index at 405 nm	Crystal 1.4 + 3.2j	Amorphous 2.8 + 2.55j	Crystal 3.5 + 4.5j	Amorphous 3.6 + 3.9j	Crystal 2.863 + 3.120j	Amorphous 1.668 + 0.309j
Reset time (ns)	50		35		20	

### Performance of massively parallel direct writing and demonstration of nano aperture fabrication on multi-layered PCMs

**Fig. 4:**
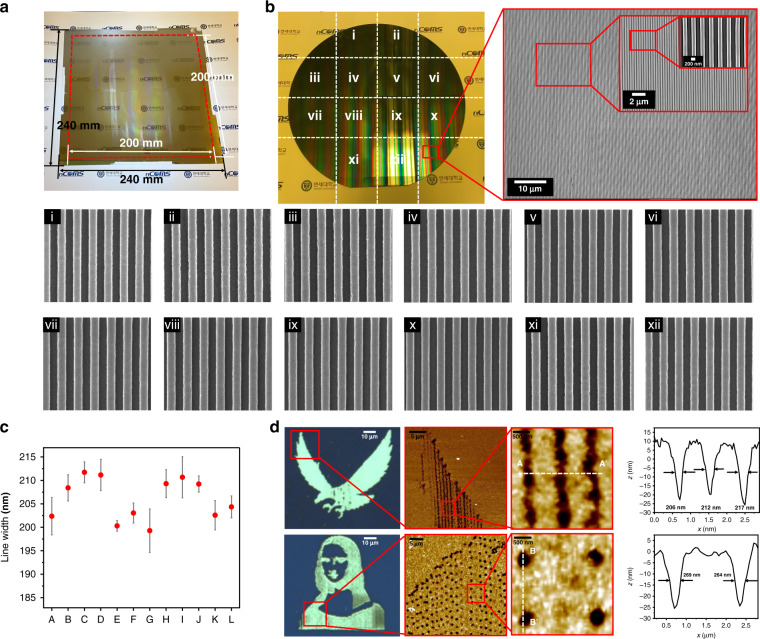
Performance of massively parallel direct writing system. **a** Results of a large-area (200 × 200-mm) nanoaperture. **b** Photo and scanning electron microscopy images of a photoresist nanopattern fabricated on an 8-inch wafer via near-field nanolithography based on the large-area nanoaperture. Pattern-uniformity results: high-resolution scanning electron microscopy image of 12 parts of the total pattern area. Six points were measured in each part (Table S2). **c** Graph of pattern-uniformity results. The mean line width of the nanopatterns was 206.04 nm, the standard deviation was 5.06 nm and the uniformity (2*σ*) was 5.22% **d** Optical microscopy and atomic force microscopy images: the eagle symbol of Yonsei University with 210-nm line patterns and the Mona Lisa with 250-nm dot patterns.

To verify the nanoapertures fabricated using this massively parallel direct-writing system, we created a large-area (200 × 200 mm) photoresist pattern through near-field lithography using an i-line commercial photolithography system (Fig. 4a, b). To determine pattern uniformity, we analyzed 12 parts of the total pattern area via high-resolution scanning electron microscopy. The mean line width of the nanopatterns was 206.04 nm, standard deviation was 5.06 nm and the uniformity (2*σ*) was 5.22% (Fig. 4b, c and Table S2). To verify the application of the massively parallel direct-writing system, nanoapertures forming the eagle symbol of Yonsei University and the Mona Lisa were developed (Fig. 4d). The line width was measured via atomic force microscopy. Moreover, the constellation for each month was formed, demonstrating that free shapes made of dots and lines can be directly patterned, as well as a photoresist with 100-nm line patterns (Figs. S6 and S7). This shows that the proposed massively parallel direct-writing system, with a multi-optical probe system and super-resolution near-fields, can create various line and dot patterns and freeform patterns. Furthermore, this method shows great potential for high-density, high-throughput lithography applications.

## Discussion and conclusion

We demonstrated a massively parallel direct-writing system through combination of a glass micro-FZP array and a multi-layered PCM. To validate the massively parallel direct-writing process, we fabricated photoresist nanopatterns via near-field nanolithography using nanoapertures created by our system. Nanoaperture fabrication on chalcogenide materials through massively parallel direct writing can enhance resolution via the near-field effect while overcoming the limitations of conventional large-area lithography techniques for nanoaperture fabrication. Compared to technologies such as E-beam and Foucusion beam, it is insufficient in terms of resolution, it is much more advantageous in terms of throughput because patterning is possible with 20×20 probes at a speed of tens of mm/s. Also, it has similar resolution and speed compared to laser interference lithography. It is possible to produce arbitrary patterns that cannot be produced by laser interference lithography. Moreover, it has a resolution 10 times better than conventional direct laser writing technology. Unlike the scanning-probe method, the massively parallel direct-writing technique supports reproduction using commercial photo equipment, enabling high speed, large-area nanopattern production and high productivity. However, the limitation of near-field lithography through nano aperture using this system uses the conventional contact photolithography system’s hard contact mode. Therefore, it was difficult to overcome the limitations of the conventional photolithography system caused by the generated particles. If these issues are well controlled, it is expected that they can develop into near-field lithography that can be used as future technologies.

## Methods

### Process for fabricating the glass FZP array

The glass thermal-imprinting process included the following steps: preheating, delivery of pressure and laser irradiation, cooling, and demolding^32^. During preheating, the heating blocks (upper and lower), glass, and silicon mold were heated to 320 °C below the glass transition temperature (*T*_g_), to prevent damage to the glass due to rapid temperature changes. We used a 2 mm-thick 10 × 10 mm K-PG375 glass (*T*_g_ 344 °C). The silicon mold and glass were in contact and subjected to pressure. When an appropriate pressure was attained, the sample was irradiated with an expanded laser beam for 30 s at 30 W, and the glass (100 mm^2^) received 628.76 J/cm^2^ of energy. A 10.6 µm infrared laser beam was passed through the silicon mold to raise the glass surface temperature above *T*_g_. The mold cavity was filled with softened glass material, cooled, and demolded. The time required to raise the glass temperature from room temperature to an appropriate preheating temperature (320 °C) was 26–27 s, and the optimal laser irradiation time was 30 s. The glass returned to room temperature within ~25 s. The cycle time (from loading of the glass substrate to demolding) was short (~83 s).

### Deposition of multi-layered PCM

The multi-layered PCM consists of phase-change material layers, dielectric layers, substrate. Quartz glass was also used as substrates, Sb_65_Se_35_ was used as phase-change material, and zinc sulfide-silicon dioxide (ZnS-SiO_2_) was used for the dielectric layers. The cleaning process for the substrates glass was achieved using an ultrasonic cleaner for 15 min with alcohol and DI water, and they were dried with an air gun. The multi-layered PCM was fabricated using an E-beam evaporation system (Korea Vacuum Tech Co.) The chamber pressure was maintained under 7 × 106 mbar for optimal thin film deposition conditions, and the deposition speed and temperature were maintained using a microbalance and Knudsen cell. ZnS-SiO_2_, Sb_65_Se_35_ were deposited at 0.5 and 0.5 Å/s, respectively.

### Fabrication process of the multi-layered PCM

The experimental set-up for multi-layered PCM writing consisted of four parts: laser control, staging, beam shaping, and beam alignment. A 405-nm diode laser was used, operating in multimode with 10 W maximum power (MDL-XD-405, CNI Co., Ltd, Changchun, China); the laser was capable of pulse modulation up to 100 MHz via a function generator. The pulse signal was generated with a function generator (AFG30152, Tektronix Inc., Beaverton, OR, USA). Pulse dynamics were confirmed with an oscilloscope (Wavelet 324; Teledyne LeCroy, Thousand Oaks, CA, USA). A 2-axis-driven air bearing stage (A-322 PIglide HS Air Bearing Stage, Physik Instrumente (PI) GmbH & Co. KG, Karlsruhe, Germany) was used to move the multi-layered PCM. For the beam alignment part, a CCD image sensor (CM-140 MCL, JAI Ltd., Copenhagen, Denmark), an objective lens of ×20 magnification, and a tube lens of ×1 magnification were used.

## Supplementary information

supplementary information

Graphical Abstract
