# Supplementary material for: Massively parallel direct writing of nanoapertures using multi-optical probes and super-resolution near-fields
Source: Microsyst Nanoeng. 2022 Sep 15;8:101. doi: 10.1038/s41378-022-00416-9 (PMC9475023; doi:10.1038/s41378-022-00416-9)
Supplement: Supplementary file 1 — supplementary information [file 41378_2022_416_MOESM1_ESM.pdf]

Supplementary Information

Massively parallel direct writing of nanoapertures using multi-optical probes and super-resolution near fields

Changsu Park[1,a,b], Soobin Hwang[1,a,b], Donghyun Kim[a,b], Nahyun Won[a,b], Runjia Han[a,b], Seonghyeon Jeon[a,b], Wooyoung Shim[c], Jiseok Lim[d], Chulmin Joo[a,b] and Shinill Kang[a,b,*]

[a]School of Mechanical Engineering, Yonsei University, 50 Yonsei-ro, Seodaemun-gu, Seoul, 03722, Korea

[b]National Center for Optically-assisted high precision Mechanical Systems, Yonsei University, Seoul, 03722, Korea

[c]Department of Materials Science and Engineering, Yonsei University, 50 Yonsei-ro, Seodaemun-gu, Seoul, 03722, Korea

[d]School of Mechanical Engineering, Yeungnam University, 280 Daehak-ro, Gyeongsan, Gyungbuk, 38541, South Korea

[1] C. Park and S. Hwang have contributed equally to this work

*Corresponding authors: snlkang@yonsei.ac.kr

Supplementary information 1: Thermal and optical properties of phase change material layer

Table S1: Properties of super resolution near-field structure

**Material**	**Refractive index**	**Thermal conductivity(J/mks)**	**Specific heat per unit mass(J/kgK)**	**Mass density(kg/m^3)**
**ZnS-SiO$_2$**	2.21	0.657	560	3650
**Sb$_{65}$Se$_{35}$**	**Crystal :** 2.863 + 3.120 i **Amorphos** : 1.668 + 0.309 i	2.575	234	5653
**PR**	1.68 + 0.0275 i	0.437	1635	1021
**Substrate (Quartz)**	1.5	1.4	1000	2200

To optimize the super-resolution film, thermal and optical simulations were conducted as described in the simulation part, and the optical and thermal properties must be measured for the simulations. The refractive index, thermal conductivity and Cp values were measured using an ellipsometer, laser flash apparatus (LFA) and differential scanning calorimetry (DSC). $Sb_{65}Se_{35}$ refractive index change was measured from an ambient temperature to 600°C using an ellipsometer, M-2000 manufactured by J. A. Woollam. For a thermal analysis of the super-resolution near-field structure, the thermal conductivity and Cp values are required; therefore, we measured these parameters using a LFA and DSC. The Cp value was measured using a DSC 200 FS system, NETZCH and the thermal conductivity was measured using a LFA 457, NETZCH.

Supplementary information 2. XRD analysis of $Sb_{65}Se_{35}$

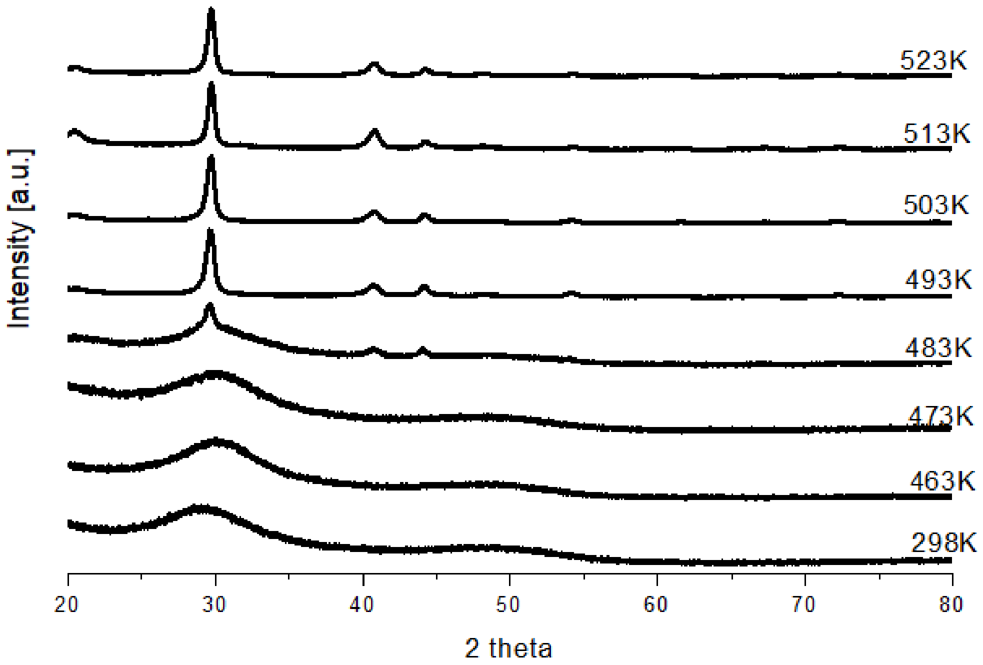

Figure S1: XRD analysis of $Sb_{65}Se_{35}$ film depending on temperature

For writing of multi-layered PCM using $Sb_{65}Se_{35}$, the corresponding layer must be deposited on the substrate and crystallized. XRD measurement was performed to determine the crystallization temperature. $Sb_{65}Se_{35}$ of 20nm was deposited on the cleaned silicon wafer using an E-beam evaporator (KVE-ENT2006; Korea Vacuum tech, Seoul, Korea), and the deposited specimens were heated on a hotplate at 10K intervals from 463 to 523K for 10 minutes. Annealed samples were measured up to 20 – 80 degrees through HR-XRD (Smartlab; Rigaku, Tokyo, Japan). The sample of 298K without separate heat treatment after deposition can be confirmed to be amorphous as two halos are observed, and as a sharp peak is generated after 483K, it can be confirmed that the temperature changes to Crystalline.

Supplementary information 3. Photo-thermal simulation according to thicknesses of upper and medium dielectric layers for varying 1st and 2nd PCM thicknesses

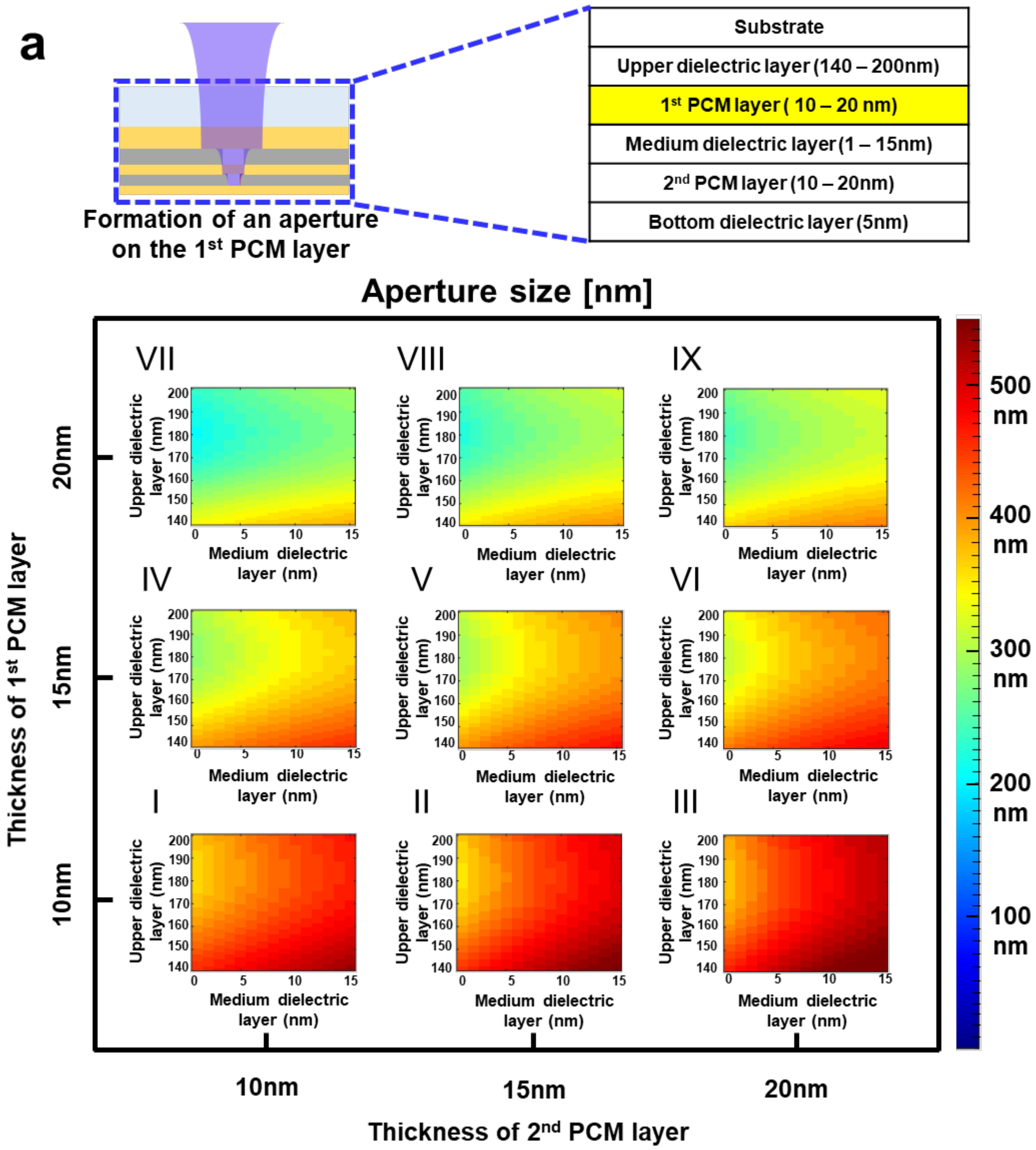

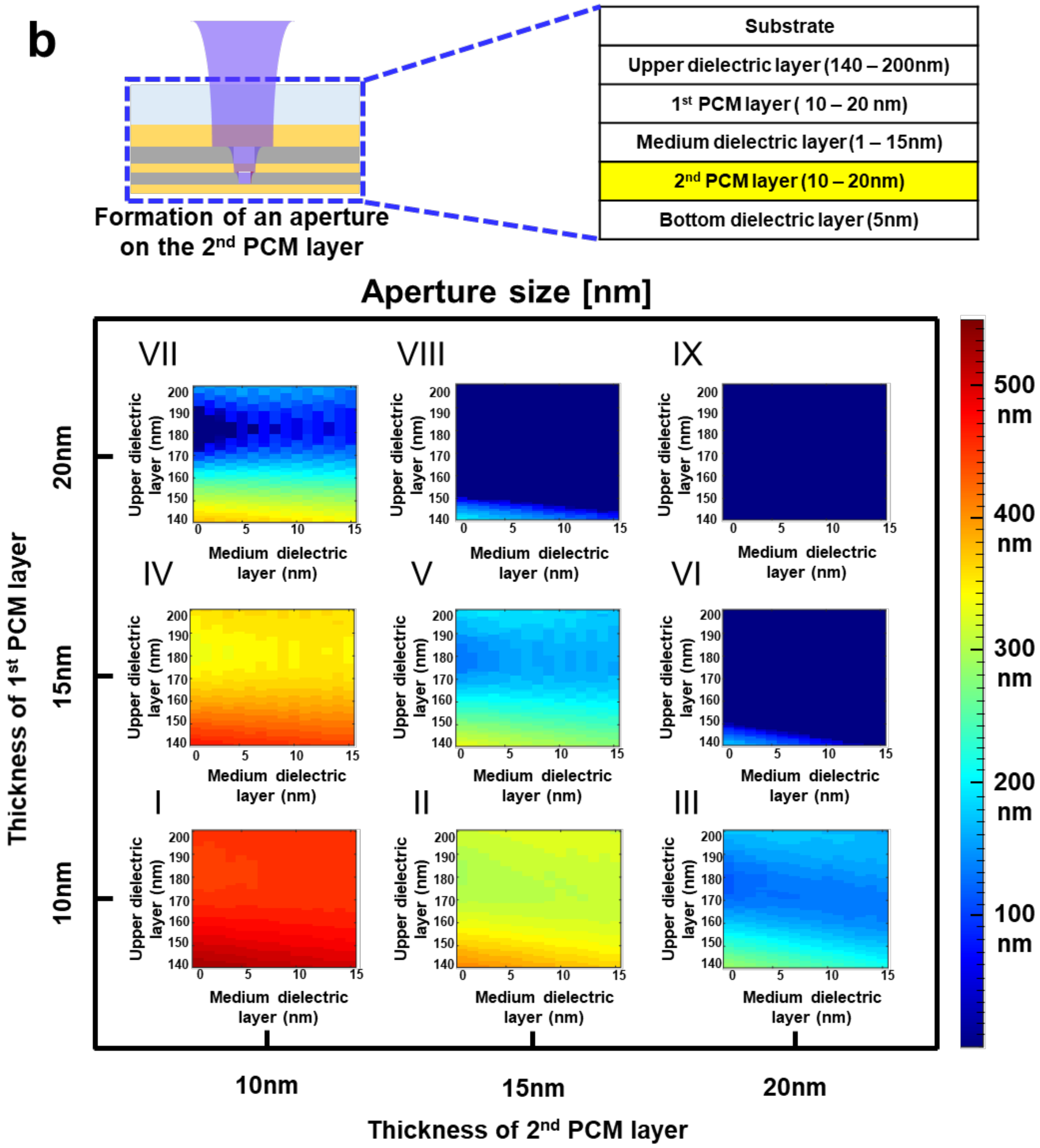

Figure S2. (a) Contour plots of aperture size on 1st PCM layer according to upper dielectric layer (140 – 200nm) and Medium dielectric layer thickness (5 – 15nm) for varying 1st and 2nd phase change layer thicknesses of (a. I) 10 – 10nm, (a. II) 10 – 15, (a. III) 10 – 20, (a. IV) 15 – 10, (a. V) 15 – 15, (a. VI) 15 – 20, (a. VII) 20 – 10, (a. VIII) 20 – 15, (a. IX) 20 – 20nm (b) Contour plots of aperture size on 2nd PCM layer according to upper dielectric layer (140 – 200nm) and Medium dielectric layer thickness (5 – 15nm)for varying 1st and 2nd PCM layer thicknesses of (b. I) 10 – 10nm, (b. II) 10 – 15, (b. III) 10 – 20, (b. IV) 15 – 10, (b. V) 15 – 15, (b. VI) 15 – 20, (b. VII) 20 – 10, (b. VIII) 20 – 15, (b. IX) 20 – 20nm.

Supplementary information 4. Photo-thermal simulation data: Comparison among chalcogenide materials ($Ge_2Sb_2Te_5$, Sb_2Te_3, $Sb_{65}Se_{35}$)

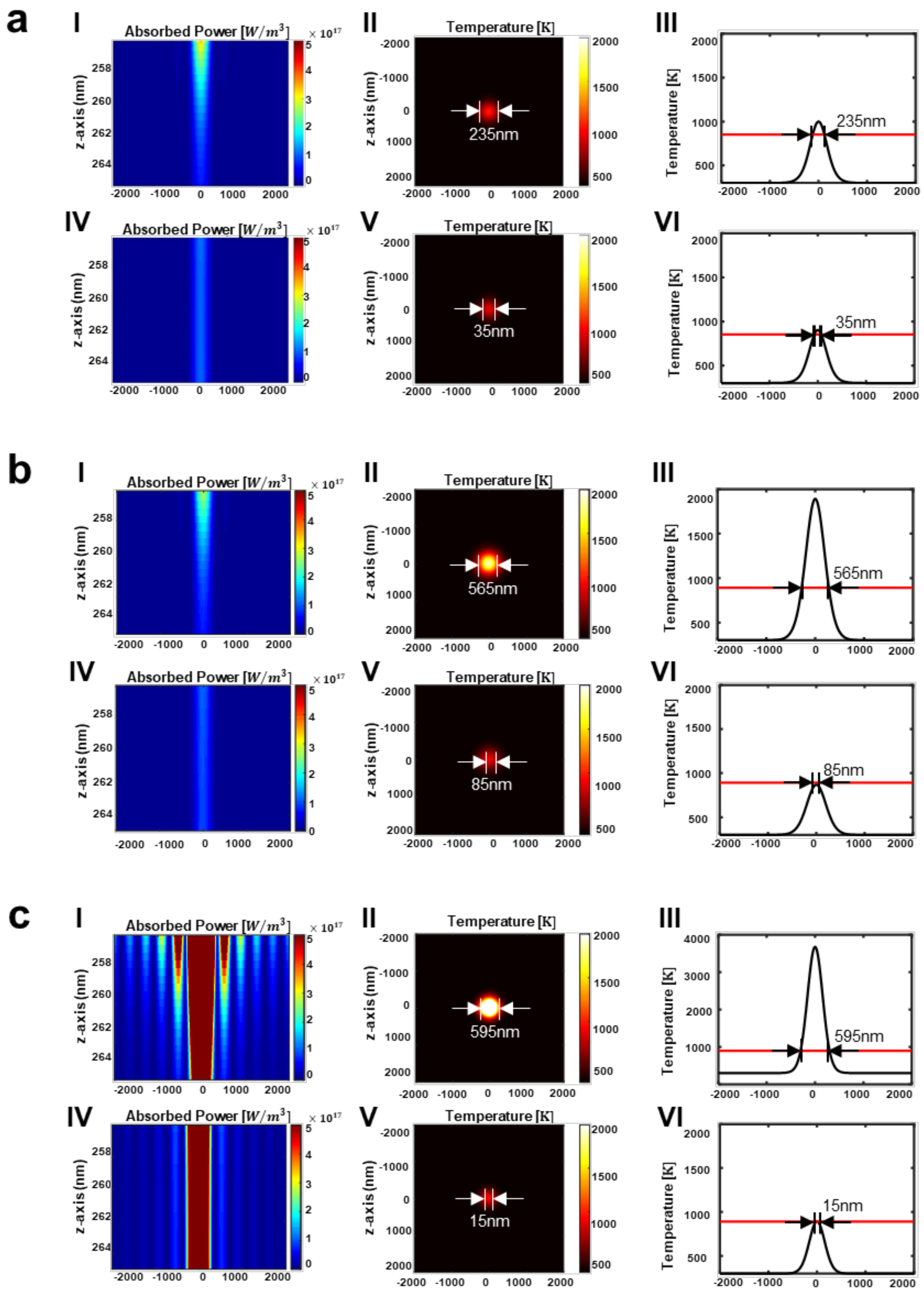

Figure S3. Aperture size prediction simulation results as functions of phase change materials: (a) $Sb_{65}Se_{35}$, (b) $Ge_2Sb_2Te_5$ (c) Sb_2Te_3 (a) analysis results of absorbed power unit volume and time(a. I, IV), temperature profile and aperture size prediction results(a. II, III, V, VI) in 1st and 2nd Sb65Se35 layer (b) analysis results of absorbed power unit volume and time(b. I, IV), temperature profile and aperture size prediction results(b. II, III, V, VI) in 1st and 2nd Ge2Sb2Te5 layer (c) analysis results of absorbed power unit volume and time(b. I, IV), temperature profile and aperture size prediction results(b. II, III, V, VI) in 1st and 2nd Sb2Te3 layer, respectively

Figure S3.a shows the power absorption of the multi-layered PCM with the optimum design and the corresponding temperature distribution. A 235-nm aperture was generated in the first PCM; a 35-nm aperture was created in the second PCM layer under 1.5 mW, 500 kHz, 20-ns pulsed laser irradiation. $Sb_{65}Se_{35}$ shows a relatively large reduction in its absorption coefficient; this determines transmission when transitioning from the crystalline to the amorphous phase. Additionally, $Sb_{65}Se_{35}$ has a smaller specific heat, and its phase change temperature is about 40 K lower compared with other PCMs for super-RENS structures, such as GST and Sb_2Te_3, as shown in Table 1.

Figure S3.b shows the numerical values for the optical aperture minimized when $Ge_2Sb_2Te_5$ material was used as multi-layered PCM. When the 1.6mW, 500Khz laser was irradiated, it was confirmed that an opening of 565 nm was formed in the 1st layer and an opening of 85 nm was formed in the 2nd layer. It was confirmed that relatively large optical apertures were formed in the 1st and 2nd layers compared to $Sb_{65}Se_{35}$, which is due to high power absorption in the 1st layer due to a higher absorption coefficient compared to $Sb_{65}Se_{35}$.

Figure S3.c shows the minimum opening size that can be obtained when the Sb_2Te_3 material is used as the material of the multi-layered PCM. In addition, it was confirmed that upon irradiating a laser beam with 60 mW, 500 kHz, apertures with sizes of 595 and 15 nm were

formed on the 1st and 2nd PCM layers, respectively. This resulted in high power absorption due to a relatively high absorption coefficient compared to $Sb_{65}Se_{35}$, but it was confirmed that the rate of temperature change was very low due to relatively high specific heat and mass density. This means that it is somewhat inadequate in terms of power efficiency to be used as a material for a multi-layered PCM.

Supplementary information 5. the composition of the $Sb_{65}Se_{35}$ in deposited multi-layered PCM

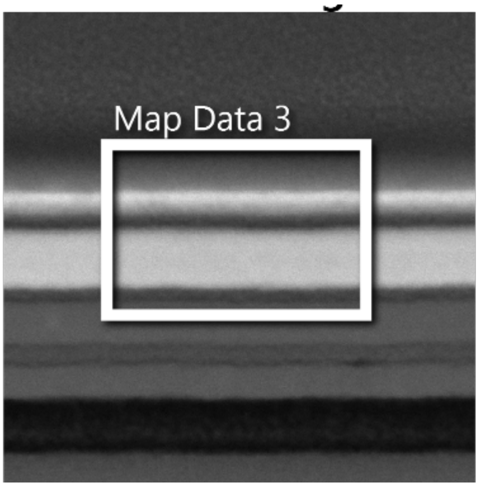

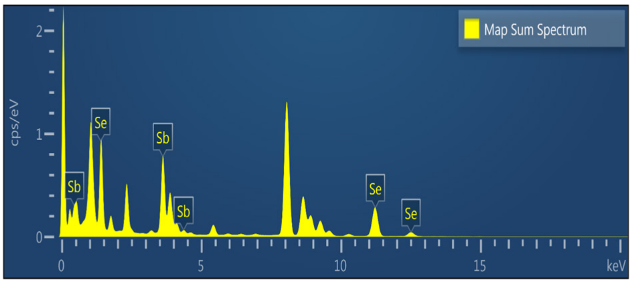

Element	Wt%	Atomic %
Se	28.36	37.90
Sb	71.64	62.10
Total:	100.00	100.00

Figure S4. TEM image and EDS mapping result to confirm the composition of the $Sb_{65}Se_{35}$ in deposited multi-layered PCM

Supplementary information 6. Uniformity of line patterns results of near-field nanolithography on 8inch wafer using large areal nano aperture

Table S2. Linewidth measurement result

	**1**	**2**	**3**	**4**	**5**	**6**
**a**	205.26	201.06	197.45	207.96	202.66	199.85
**b**	210.67	210.67	207.96	205.26	205.26	210.67
**c**	216.07	211.17	210.67	210.67	210.67	211.17
**d**	216.07	210.67	210.94	207.96	213.37	207.96
**e**	199.85	199.95	199.85	199.85	202.56	199.85
**f**	199.85	202.56	202.76	202.66	205.26	205.26
**g**	194.55	202.56	197.85	194.45	205.26	201.06
**h**	207.96	207.96	205.26	210.67	213.37	210.67
**i**	208.06	213.37	205.26	207.96	216.07	213.37
**j**	210.67	207.96	210.67	210.67	208.26	207.11
**k**	205.26	202.56	197.15	205.36	202.66	202.56
**l**	202.56	207.96	205.26	202.56	205.26	202.56

Supplementary information 7. Monthly constellation patterns.

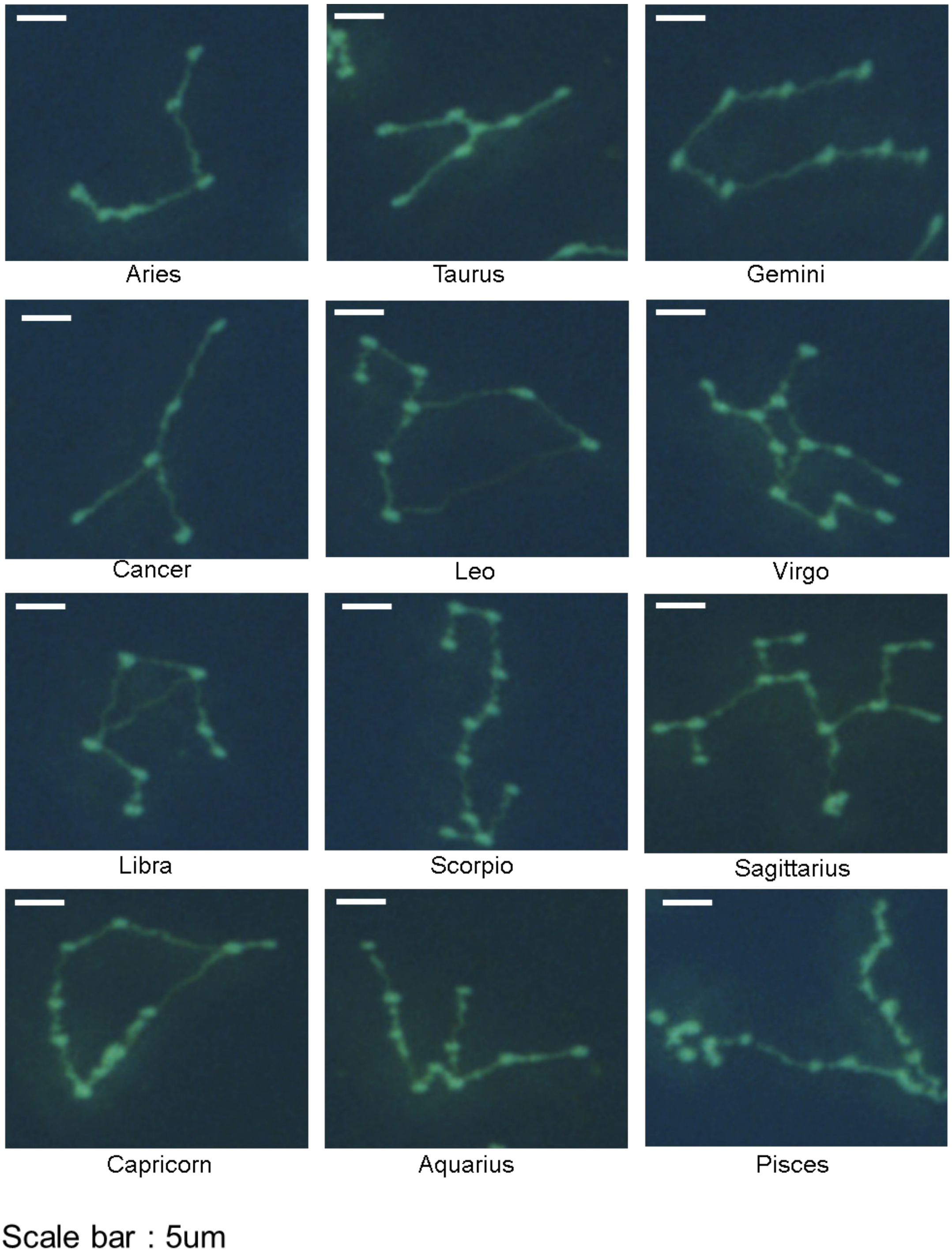

Figure S5. Optical microscopy images of monthly constellation patterns on multi layered PCM. (a) Aries, (b) Taurus, (c) Gemini, (d) Cancer, (e) Leo, (f) Virgo, (g) Libra, (f) Scorpio, (g) Sagittarius, (h) Capricorn, (i) Aquarius, (j) Pisces

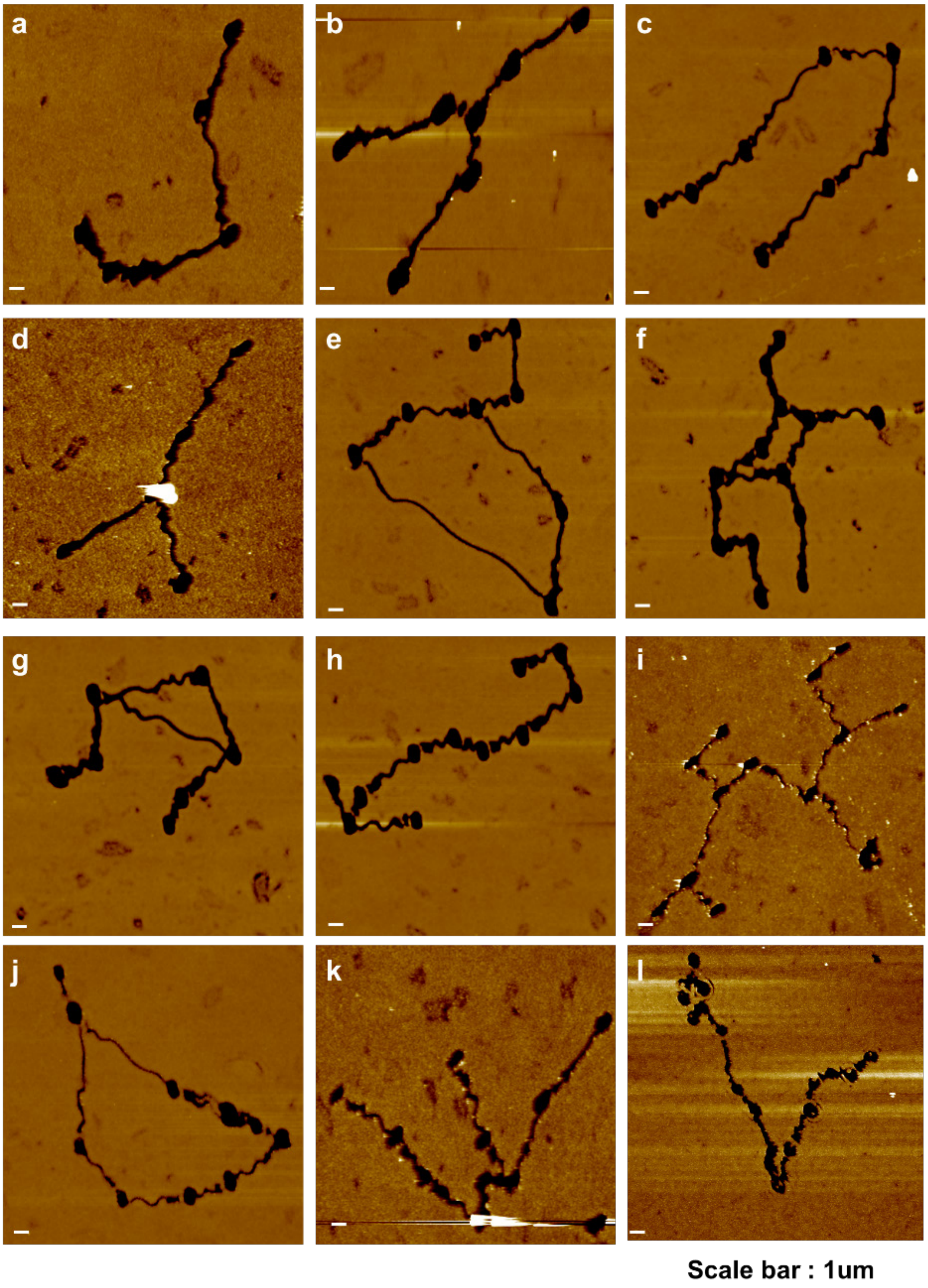

Figure S6. AFM images of monthly constellation patterns on photoresist. (a) Aries, (b) Taurus, (c) Gemini, (d) Cancer, (e) Leo, (f) Virgo, (g) Libra, (f) Scorpio, (g) Sagittarius, (h) Capricorn, (i) Aquarius, (j) Pisces
