# Supplementary figures and images for: Massively parallel direct writing of nanoapertures using multi-optical probes and super-resolution near-fields
Source: Microsyst Nanoeng. 2022 Sep 15;8:101. doi: 10.1038/s41378-022-00416-9 (PMC9475023; doi:10.1038/s41378-022-00416-9)

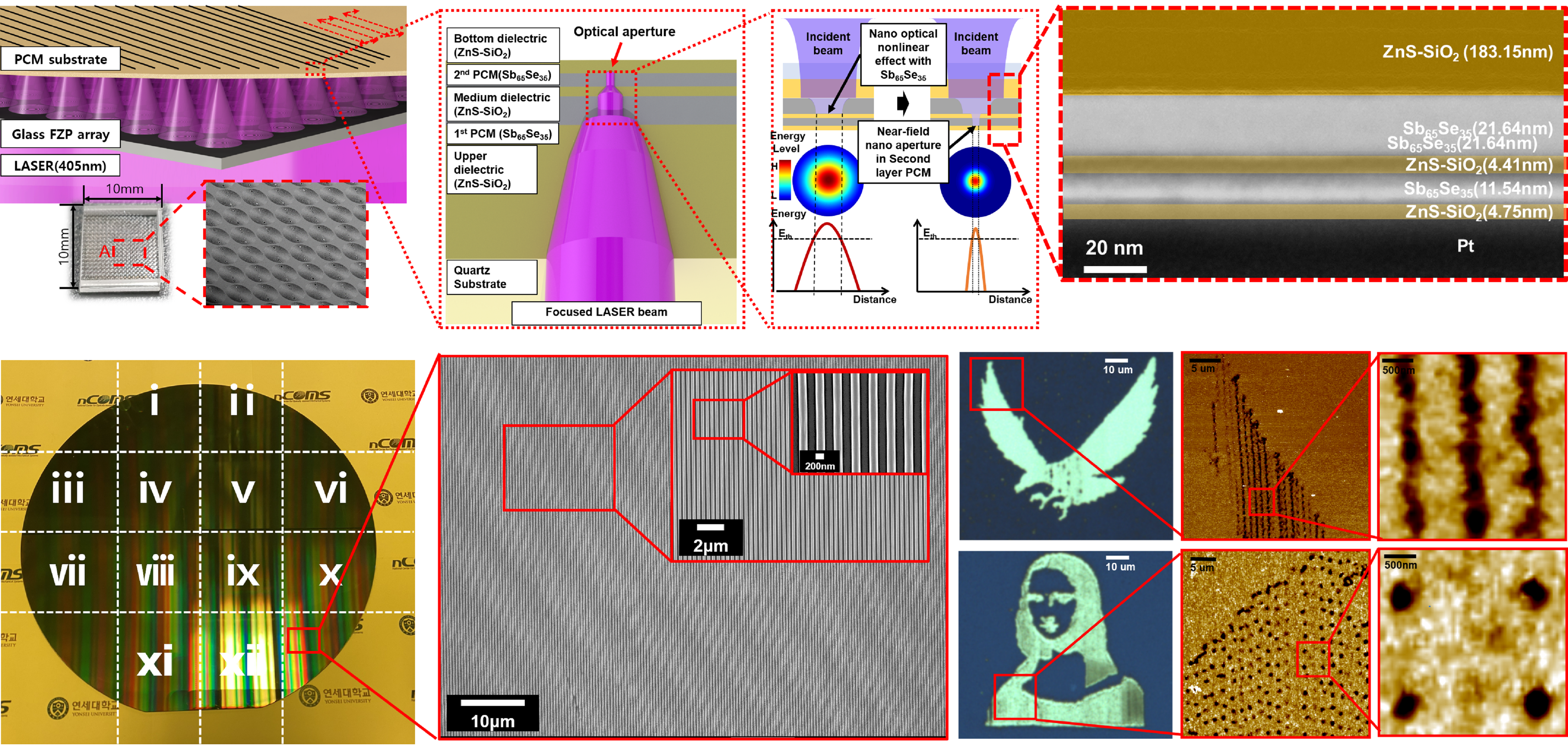

Supplement: Supplementary file 2 — Graphical Abstract [file 41378_2022_416_MOESM2_ESM.tif]
